# β-Galactosylceramidase in cancer: more than a psychosine scavenger

**DOI:** 10.18632/oncoscience.551

**Published:** 2022-03-23

**Authors:** Mirella Belleri, Marco Presta

**Keywords:** cancer, ceramide, galactosylceramidase, lipidome, sphingolipids

Sphingolipids, a class of compounds composed by a sphingoid base backbone, represent major components of biological membranes, and play a pivotal role in a variety of subcellular signaling processes. Abnormal sphingolipid metabolism sets the basis for the pathogenesis of variety of genetic diseases known collectively as sphingolipidosis, or sphingolipodystrophy. Among them, globoid cell leukodystrophy (also named Krabbe disease; OMIM #245200), is an autosomal recessive sphingolipidosis characterized by degeneration of oligodendroglia and progressive demyelination due to the genetic deficiency of *β-galactosylceramidase* (GALC; EC 3.2.1.46) [1], a lysosomal acid hydrolase that catalyzes the removal of β-galactose from β-galactosylceramide (GalCer) and other terminal β-galactose-containing sphingolipids. Based on a long-held and recently confirmed “psychosine hypothesis” [2], Krabbe disease may manifest as a consequence of the accumulation of the neurotoxic GALC substrate β-galactosylsphingosine (psychosine) in the central and peripheral nervous system [3]. Thus, most of the studies concerning the biological role of GALC have been performed on Krabbe patients and *Galc*-deficient *twitcher* mice (an authentic animal model of the disease [4]), leading to the envision that the major biological function of GALC may consist in its psychosine “scavenging” activity.

However, experimental evidence indicates that GALC may act not only as a psychosine scavenger, but its modulation also exerting a series of psychosine-independent effects [5, 6]. For instance, GALC deficiency affects neovascularization in *in vitro* and *in vivo* in the presence of negligible, if any changes in psychosine levels [7, 8]. In addition, knock-down of the human GALC ortholog *galcb* in zebrafish embryos affects cell survival and neuronal differentiation in the absence of any significant accumulation of this metabolite [9]. In this frame, a recent study has shown that *Galc* knock-down in murine melanoma B16 cells causes a significant increase of the levels of the oncosuppressive sphingolipid ceramide mirrored by a decrease of sphingomyelins, phosphatidylethanolamines and cholesteryl esters, paralleled by an increased concentration of diacylglycerols [10]. These alterations of the lipidomic profile resulted in the inhibition of the tumorigenic activity of murine melanoma B16 cells. Increased levels of ceramide were observed also in *GALC*-silenced human melanoma A2058 cells and tumor xenografts, with a consequent decrease of their tumorigenic potential [10]. In keeping with these observations, a progressive increase of *GALC* expression occurs during tumor progression in human pathological skin specimens ranging from common nevi to stage IV melanoma. Again, the levels of *GALC* expression were inversely related to the levels of ceramide immunoreactivity in the same tumor samples [5, 10]. Together, these findings indicate that GALC might act as an oncogenic enzyme during melanoma progression by decreasing the levels of the oncosuppressive ceramide.

These data also bring up the question of the mechanisms responsible for the observed inverse relationship that occurs between GALC activity and ceramide levels in melanoma. In this frame, we have observed that GALC down-regulation in murine and human melanoma cells leads to a non-redundant upregulation of *sphingomyelin phosphodiesterase 3* (*Smpd3*) [10]. This gene encodes for neutral sphingomyelinase 2, an oncosuppressive enzyme that catalyzes the hydrolysis of sphingomyelin to form phosphocholine and ceramide ([11] and references therein). Accordingly, SMPD3 immunoreactivity decreases in human melanoma specimens during tumor progression, in parallel with the observed decrease of ceramide levels and *GALC* upregulation [10]. Thus, it seems possible to hypothesize that GALC may suppress ceramide synthesis by inducing the downregulation of *SMPD3* expression. Further studies are required to elucidate the mechanism(s) at the basis of the GALC/SMPD3 cross talk and its impact on ceramide metabolism.

Notably, the pro-oncogenic activity of GALC might not be limited to melanoma. Indeed, as stated above, GALC activity appears to play a non-redundant role in angiogenesis, a hallmark of cancer [7, 8]. In addition, high levels of immunoreactive GALC are associated with poor prognosis in colorectal cancer and *GALC* expression in circulating tumor cells correlates with the presence of distant metastases and poor response to therapy in lung cancer patients ([5, 6] and references therein).

It should be pointed out that GALC may exert also oncosuppressive effects in some tumor types, as discussed in [5, 6]. Briefly, scattered experimental observations indicate that *GALC* expression is downregulated in a panel of head and neck and of lung cancer cell lines and in Epstein-Barr virus-associated nasopharyngeal carcinoma. This downregulation appears to be the consequence of CpG island hypermethylation of the *GALC* gene promoter [5]. In these cases, it seems possible to hypothesize that GALC may act as an oncosuppressor by increasing the levels of ceramide derived by GalCer hydrolysis, GALC downregulation leading to a decrease of ceramide that may allow tumor progression.

**Figure 1:**
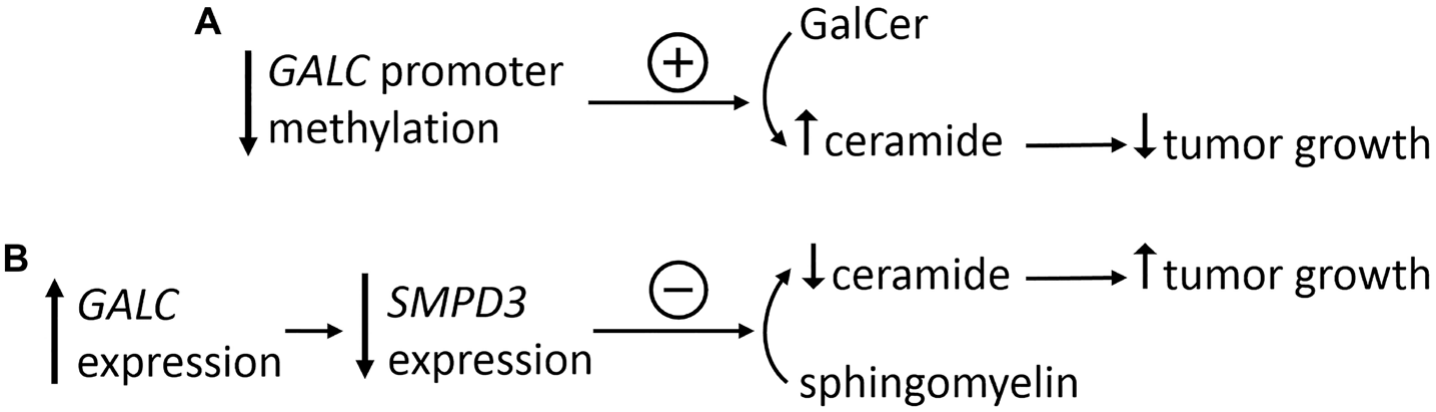
Impact of GALC expression on tumor growth. (**A**) *GALC* promoter hypomethylation causes an elevated production of the enzyme that leads to increased levels of the oncosuppressor ceramide starting from its GalcCer precursor, with a consequent decrease of tumor growth. (**B**) Increased levels of *GALC* expression causes the downregulation of *SMPD3* with a consequent decrease of sphingomyelin to ceramide conversion, leading to an increase of tumor growth.

The contrasting findings about a dual role of GALC in cancer progression suggest that this sphingolipid-metabolizing enzyme may exert both oncosuppressive and oncogenic functions in tumor biology depending on its negative or positive impact of the intracellular concentration of ceramide, mediated at least in part by the effect of GALC on the expression of the ceramide-producing enzyme SMPD3 (Figure 1). At present, no data are available to predict in which tumor type, clinical and/or experimental condition GALC may exert an oncogenic or oncosuppressive activity. Nevertheless, these observations indicate that the envision of GALC as a mere “psychosine scavenger” ignores the possibility that this enzyme may exert a wider impact in tumor biology. In this frame, studies about the effects of the modulation of GALC activity on tumor lipidome are eagerly required. These studies will allow a better understanding of the role of GALC in tumors and of its clinical implications in anticancer therapy.
